# Proceedings of the Tenth International Meeting on Neuroacanthocytosis Syndromes

**DOI:** 10.5334/tohm.622

**Published:** 2021-05-21

**Authors:** Merc Masana, Manuel J. Rodrguez, Jordi Alberch

**Affiliations:** 1 Dept. Biomedical Sciences, Institute of Neurosciences, School of Medicine and Health Sciences, Universitat de Barcelona, Barcelona, Spain; 2 Institut dInvestigacions Biomdiques August Pi i Sunyer (IDIBAPS), Barcelona, Spain; 3 Centro de Investigacin Biomdica en Red sobre Enfermedades Neurodegenerativas (CIBERNED), Madrid, Spain

**Keywords:** neuroacanthocytosis, acanthocytosis, McLeod syndrome, chorea-acanthocytosis, VPS13A

## Abstract

The 10th International Meeting on Neuroacanthocytosis Syndromes was held online on March 10th12th, 2021. The COVID19 pandemic situation made our planned meeting in Barcelona on March 2020 to be suspended by one year, and finally took place online. The meeting followed the previous nine international symposia, the last of which was held in Dresden, Germany in March, 2018. The setting of the meeting encouraged interactions, exchange of ideas and networking opportunities among the high number of participants from around the globe, including scientists, neurologists and specially patients and caregivers. A total of 27 oral communications were distributed in 8 sessions with topics ranging from molecular and cellular functions of VPS13 genes and proteins, their involvement in Neuroacanthocytosis Syndromes and finally clinical aspects and patients care. In addition, 5 posters were presented. Altogether, scientists and neurologists discussed recent advances and set the bases for next steps, action points, and future studies in close collaboration with the patients associations, which are always actively involved in the whole process.

## Introduction

Neuroacanthocytosis syndromes are rare disorders that include acanthocytosis of red blood cells, and neurological manifestations such as chorea, epilepsy, and problems with mood, thinking and memory. Mutations in the *VPS13A* and *XK* genes cause chorea-acanthocytosis (ChAc) and McLeod syndrome, respectively. These two syndromes were the focus of most of the talks during the meeting, although the involvement of other VPS13 genes in several VPS13opathies were also presented and discussed. Detailed clinical phenotyping, genetic analysis, Western blot diagnosis (in the case of VPS13A) and biomarkers such as creatine kinase (CK) elevation and red blood cell sedimentation, emerged as key elements that might be used to detect, diagnose and follow progression of the syndromes. Common cellular and molecular mechanisms between the different VPS13opathies were present throughout the meeting. These include the role of the different VPS13s in mediating lipid transport in membrane contact sites, as well as their involvement in mitochondria, endoplasmic reticulum, lipid droplet and endosomal functions. Direct interactions between VPS13A and XK proteins may explain some of the common features between ChAc and McLeod syndrome. Also, the relation between VPS13A function and Lyn, PIP3 and IDH3A signaling, together with calcium modulation in the cell may provide potential therapeutic targets for ChAc treatment. Finally, recent insights in VPS13A distribution in the mouse brain, and advances in the phenotyping of mouse models of ChAc, were described and might pave the way for improved understanding ChAc pathophysiology.

At the therapeutic level, outcomes from pharmacological targeting of Lyn kinase and PI3K pathway were detailed, and indicated positive effects in treating red cell blood alterations in patients but not central nervous system symptoms. Deep brain stimulation of globus pallidus was suggested as palliative treatment for patients. Because of the low number of patients worldwide, sharing information regarding patients was encouraged and, in this regard, improvements on the neuroacanthocytosis case registry database were discussed, with the aim of re-activating its use and ensure its continuity for the benefit of all the community. Importantly, patients and caregivers received professional advices for improving their respective quality of lives.

As with our recent symposia, basic scientists and clinicians discussed recent advances and set the bases for next steps, action points, and future studies, in close collaboration with the patient associations, which are always actively involved.

## New developments in translational research

### New developments in translational research

Jordi Alberch^1,2,3^, Manuel J Rodrguez^1,2,3^ and Merc Masana^1,2,3^

1. Dept. Biomedical Sciences, Institute of Neurosciences, School of Medicine and Health Sciences, Universitat de Barcelona, Barcelona, Spain
2. Institut dInvestigacions Biomdiques August Pi i Sunyer (IDIBAPS), Barcelona, Spain
3. Centro de Investigacin Biomdica en Red sobre Enfermedades Neurodegenerativas (CIBERNED), Madrid, Spain.

#### Abstract

Twenty years ago, it was discovered a conserved sorting-associated protein (VPS13A) that was mutant in chorea-acanthocytosis (ChAc). The lack of this protein triggers a cascade of intracellular events leading to selective neurodegeneration, mainly in the brain. However, the molecular and cellular function of VPS13 proteins only recently start to emerge. VPS13 is a peripheral membrane protein that transfer lipids between organelles, and it is localized at membrane contact sites between endoplasmic reticulum, vacuole, endosome and mitochondria. The mutations in VPS13 can produce also several intracellular defects such as actin polymerization, autophagy, and mitochondria integrity. Interestingly, it has been recently described that dysregulation of a VPS13A-XK complex is the common basis for ChAc and McLeod Syndrome.

In spite of these studies about the function of VPS13A, little is known about the function of this protein in the nervous system and why this mutation is affecting specific neuronal populations. In mouse brain, VPS13A is predominantly localized to microsomal and synaptosomal fractions, neuronal perinuclear region, cytoplasm, and fibers. In any case, the expression levels and subcellular localization of the protein cannot explain the specific neuronal vulnerability. Interestingly, recent studies have provided new data thanks to the differentiation of iPSCs generated from skin biopsies of ChAc patients, describing mechanisms involved in neuronal survival and calcium homeostasis. Interestingly, a recent study using a transcriptomic analysis showed that VPS13A is involved in tolerance to cocaine-induced motor disturbances, with a differential regulation between the dorsal and ventral striatum. Thus, next step must be focused to study the effect of the lack of VPS13A in the brain to understand how the mutation can affect neuronal connectivity between the different neuronal populations and the mechanisms that control the selective vulnerability.

## Neuroacanthocytosis syndromes and VPS13opathies

### Neuroacanthocytosis syndromes and VPS13opathies: the pathophysiological spectrum

Andreas Hermann^1,2,3^

1. Translational Neurodegeneration Section Albrecht-Kossel, Department of Neurology, University Medical Center Rostock, University of Rostock, Rostock, Germany.
2. DZNE, German Center for Neurodegenerative Diseases, Research Site Rostock/Greifswald, Rostock, Germany.
3. Center for Transdisciplinary Neurosciences Rostock (CTNR), University Medical Center Rostock, University of Rostock, Rostock, Germany.

#### Abstract

Chorea-Acanthocytosis (ChAc) is one of the core neuroacanthocytosis (NA) syndromes characterized by a movement disorder and blood cell acanthocytosis. The other main NA syndrome is McLeod syndrome with mutations in the XK gene, besides rare variants of NA such as Pantothenate kinase-associated neurodegeneration (PKAN), Huntington disease-like 2 or Abeta-/hypolipoproteinaemia syndromes besides others. This clinically associated syndromes originate from very different genetic alterations.

ChAc is an ultra-rare neurodegenerative disease caused by mutations in the VPS13A gene (vacuolar protein-sorting protein 13). VPS13A belongs to a gene family of four members (VPS13 A-D), for which only one orthologue exist in yeast (vsp13). This prompted speculations since many years about the other VPS13opathies also possibly being associated to neurological diseases. Mutations in VPS13B (COH1) are known to cause Cohen syndrome, a developmental disorder with intellectual disability and distinct craniofacial abnormalities. Recent developments identified VPS13C and D as disease causing mutations in other movement disorders, namely mutations in VPS13C cause autosomal recessive Parkinsons disease/Lewy body disease and VPS13D mutations cause spinocerebeller ataxia with saccadic intrusions (SCAR4).

Pathophysiologically, mammalian VPS13 A-D family members descend from one vps13 orthologue in yeast. Also mechanistically, there are somehow connected in cellular (patho-) physiology. While affecting different organelles and different proteins, they all are more or less connected to lipids and membrane shuttling within or between organelles.

The talk will give current insights in the clinical and molecular diversity and overlap on the VPS13inopathies and suggests that the genetic/molecular classification towards VPS13inopathies rather than to neuroacanthocytosis syndromes might be helpful in developing future (molecular) disease modifying treatments.

### Clinical aspects of chorea-acanthocytosis and McLeod syndrome

Ruth H. Walker^1,2^

1. Department of Neurology, James J. Peters Veterans Affairs Medical Center, Bronx, NY, USA.
2. Department of Neurology, Mount Sinai School of Medicine, New York City, NY, USA.

#### Abstract

Neuroacanthocytosis syndromes are characterized by the presence of acanthocytes abnormally contracted red blood cells with thorny protrusions, in addition to neurological symptoms. These inherited disorders involve basal ganglia neurodegeneration and typically present with a spectrum of movement disorders, including chorea, dystonia, tics, and parkinsonism. A range of psychopathologic features can be seen, including depression, psychosis, cognitive impairment, disinhibition, and obsessive-compulsive behaviors. Neurologic signs which distinguish these disorders from other basal ganglia conditions include seizures and peripheral neuropathy. The typical rubber person gait is due to a combination of dystonia, neuropathy, and probably motor impersistence. Dysarthria can be prominent and very disabling. Dysphagia requires ongoing management to minimize risks of aspiration pneumonia. Elevated creatine kinase and liver enzyme abnormalities can suggest either diagnosis. Neuroimaging demonstrates atrophy of the caudate nucleus and putamen. Acanthocytes are variably present and can be absent. Chorea-acanthocytosis is an autosomal recessive syndrome caused by mutations of the gene encoding for the protein chorein. Patients typically present with a combination of symptoms in young adulthood which slowly progress over 12 decades. Prominent orofacial dyskinesia and swallowing dystonia suggests the diagnosis, as does self-mutilating tongue- and lip-biting. The diagnosis can be made by Western blot demonstrating the absence of the protein in peripheral blood. McLeod syndrome is an X-linked recessive disorder, caused by mutations of the gene encoding for the McLeod protein XK. Cardiomyopathy and hepatosplenomegaly are distinguishing features; the former should be annually monitored to reduce complications. Presentation is usually in middle age, and can be quite variable, with a slow rate of progression. The diagnosis is made by antigen profiling of the erythrocyte, demonstrating absent expression of XK and decreased expression of Kell antigens.

### Bloody jello: Colloidal physics explains acanthocyte sedimentation rate as a diagnostic biomarker for neuroacanthocytosis

Alexis Darras^1^, Kevin Peikert^2,3^, Antonia Rabe^1,4^, Franois Yaya^1,5^, Greta Simionato^1,6^, Thomas John^1^, Anil Kumar Dasanna^7^, Semen Buvalyy^7^, Jrgen Geisel^8^, Andreas Hermann^2,3,9,10^, Dmitry A. Fedosov^7^, Adrian Danek^11^, Christian Wagner^1,12^ and Lars Kaestner^1,4^

1. Experimental Physics, Saarland University, 66123 Saarbruecken, Germany.
2. Translational Neurodegeneration Section Albrecht-Kossel, Department of Neurology, University Medical Center Rostock, University of Rostock, Rostock, Germany.
3. Neurodegenerative Diseases, Department of Neurology, Technische Universitt Dresden, Dresden, Germany.
4. Theoretical Medicine and Biosciences, Saarland University, 66424 Homburg, Germany.
5. Laboratoire Interdisciplinaire de Physique, UMR 5588, 38402 Saint Martin dHres, France.
6. Institute for Clinical and Experimental Surgery, Saarland University, 66424 Homburg, Germany.
7. Institute of Biological Information Processing and Institute for Advanced Simulation, Forschungszentrum Jlich, 52425 Jlich, Germany.
8. Institute for Clinical and Experimental Surgery, Saarland University, 66424 Homburg, Germany.
9. DZNE, German Center for Neurodegenerative Diseases, Research Site Rostock/Greifswald, Rostock, Germany.
10. Center for Transdisciplinary Neurosciences Rostock (CTNR), University Medical Center Rostock, University of Rostock, Rostock, Germany.
11. Neurologische Klinik und Poliklinik, Ludwig-Maximilians-Universitt, 81366 Munich, Germany.
12. Physics and Materials Science Research Unit, University of Luxembourg, Luxembourg City, Luxembourg.

#### Abstract

**Background:** Chorea-acanthocytosis (ChAc) and McLeod syndrome (MLS) are the core diseases of rare congenital neurodegenerative disorders summarized as neuroacanthocytosis syndrome (NAS). NAS patients have an increased number of irregular spiky erythrocytes, so-called acanthocytes. The detection of acanthocytes is error-prone and often leads to misdiagnosis of NAS.

**Methods:** Blood samples were collected from 6 ChAc, 3 MLS patients and 8 healthy donors. The standard Westergren method was used to record detailed erythrocyte sedimentation rate (ESR) curves. To address the mechanism involved we (i) manipulated ESR conditions (dextran- and saline solutions, plasma swap), (ii) performed optical investigations (e.g., 3D-confocal microscopy, aggregation force measurements with holographic optical tweezers) and (iii) performed theoretical modelling.

**Results:** The 2h ESR distance of 10 mm is a threshold to differentiate NAS patients and healthy donors without overlap. Colloidal physics provides a mechanistic explanation with the hole size of the percolating network (patients: 5.67 0.57 m vs. control: 8.53 1.53 m; p = 0.0045) as a characteristic value. Both erythrocytes and plasma properties contribute to the prolonged ESR. The plasma of patients has lower fibrinogen level (patients: 233 38 mg/dl vs. control: 303 46 mg/dl; p = 0.0034) resulting on lower aggregation forces (1.16 0.97 pN vs. 4.79 1.56 pN; p = 0.0005).

**Conclusion:** The ESR is a clear and robust diagnostic marker for ChAc and MLS that can easily be integrated into the standard diagnosis of patients with neurodegenerative symptoms. In addition, this study is a hallmark of the physical view of the erythrocyte sedimentation process by describing anticoagulated blood in stasis as a percolating gel, allowing the application of colloidal physics theory.

### McLeod Syndrome an overview

Hans H Jung^1^

1. Department of Neurology, University Hospital Zurich, Switzerland.

*hans.jung@usz.ch*

#### Abstract

McLeod syndrome (MLS) is a relentlessly X-linked progressive neurodegenerative disorder characterized by a progressive choreatic movement disorder and possible psychiatric as well as cognitive manifestations. Additional manifestations include peripheral neuropathy, neurogenic muscle atrophy, myopathy as well as cardiological involvement. Most patients have diminished and absent tendon reflexes and elevated serum levels of creatine kinase. Blood smears may demonstrate red blood cell acanthocytosis, which lead to the assumption of so-called neuroacanthocytosis syndromes, along with the VPS13A disease choreoacanthocytosis (ChAc), with which MLS shares a considerable phenotypic overlap. Diagnosis of MLS bases on the typical phenotype, presence of CK elevation and possible red cell acanthocytosis. The diagnosis is confirmed by presence of the so-called McLeod blood group phenotype (absent Kx antigen, weakened or absent Kell antigens) and molecular genetic confirmation of a pathogenetic alteration in the disease causing XK gene. Management of MLS is led by the individual symptoms. Of note, possible transfusion reactions should be respected and autologous blood donations might be attempted. In addition, regular cardiological follow-up examinations are mandatory.

## VPS13 genes in movement disorders

### Genetics of Chorea-Acanthocytosis: the example of Spanish patients

Antonio Velayos-Baeza^1,2^

1. Wellcome Centre for Human Genetics, University of Oxford, Oxford, UK.
2. Dept. of Physiology, Anatomy and Genetics, University of Oxford, Oxford, UK.

#### Abstract

Chorea-Acanthocytosis (ChAc), one of the core Neuroacanthocytosis syndromes, is an adult-onset rare neurodegenerative disorder caused by alteration in gene VPS13A, one of the four members of the human gene family presenting similarity to yeast VPS13 gene; all of these genes have now been associated with recessive disorders.

Extensive screening in affected patients revealed ChAc results from a wide range of pathogenic variations, including large deletions/duplications, splicing-site mutations, small insertions/deletions, nonsense mutations, or missense mutations, with a gene-wide distribution and no hot-spots. These studies provided important insight about this disorder, including the following. First, despite several RNA transcript variants having been described, only the major A variant, originally described as including exons 168+7073, appears to be directly associated with ChAc. Second, most pathogenic variations lead to absence of the VPS13A protein (chorein), confirming that this disease results from loss-of-function of chorein; this allowed to develop a semi-diagnostic test based on the analysis by western blotting of protein extracts from blood, an approach much faster and cheaper than DNA sequencing. Third, a relatively small number of the described pathogenic variations are missense mutations, suggesting that some of the affected residues might be particularly relevant for the function or the stability of chorein. Fourth, despite a number of reports about the occurrence of dominant ChAc (patients presenting with ChAc syndrome but having only one VPS13A allele mutated), the current scientific data only support the recessive nature of this condition.

The above points will be among those discussed in a presentation about the current knowledge on the genetics of ChAc in general. As an example to illustrate some of these points, specific details will be presented on the genetics of Spanish patients, one of the largest groups of studied ChAc cases, in which many of the different types of pathogenic variations have been found.

### Chorein Western blot diagnosis and VPS13A mutation searching

Gabriel Miltenberger-Miltenyi^1^

1. Department of Neurology, Ludwig-Maximilians-Universitt Mnchen, Munich, Germany.

#### Abstract

As of 2020, with the help of a large group of international colleagues, we have collected blood samples of more than 700 patients with a clinical suspicion of VPS13A disease (formerly chorea-acanthocytosis, ChAc) or related disorders (Danek, Bader & Miltenberger).

So far, we confirmed a diagnosis of VPS13A disease by Western blot of the chorein protein in 190 patients. Our free diagnostic chorein Western blot service is generously financed by the Advocacy for Neuroacanthocytosis patients. We have started to compare different protein detection protocols to achieve the most reliable approach for protein-based diagnosis of VPS13A disease. Results from the currently run batch of 80 samples will be presented. In parallel, we analyze the VPS13A gene to gain more information about the associations between genetic changes and chorein levels. In the first analysis of patients with normal chorein (22 cases), we have detected mutations in six patients. In four patients a core neuro-acanthocytosis syndrome was diagnosed: in two patients each from mutations in the VPS13A gene (confirming the diagnosis of VPS13A disease) and from mutations in the XK gene, associated with McLeod syndrome. Two patients showed variants in genes associated with other types of movement disorders (KMT2B, GNAL). Overall, we have currently observed six cases with known VPS13A mutations in the presence of normal appearing chorein bands in Western blotting and discuss the details in a dedicated presentation at this meeting (Miltenberger et al.). To systematically catalogue all known VPS13A mutations, we joined the widely used open access LOVD (Leiden Open Variation Database) as curators for the VPS13A gene (Miltenberger, Velayos-Baeza & Danek: *https://databases.lovd.nl/shared/genes/VPS13A*).

### The role of VPS13C and VPS13D genes in the pathogenesis of movement disorders

Coro Paisn-Ruz^1^

1. Department of Neurology, Icahn School of Medicine at Mount Sinai, New York, USA.

#### Abstract

Pathogenic genetic variations within VPS13C and VPS13D genes have recently been identified in movement disorder phenotypes. An overview of the disease-associated phenotypes and genetic variations will be summarized here to assist in the clinical diagnosis of prospective patients and families.

### Contribution of rare homozygous and compound heterozygous VPS13C missense mutations to dementia with Lewy bodies and Parkinsons disease

Stefanie Smolders^1,2,3^, Stphanie Philtjens^1,2,3^, David Crosiers^1,2,3,4^, Anne Sieben^1,2,6^, Elisabeth Hens^1,2,3,4,5^, Bavo Heeman^1,2,3^, Sara Van Mossevelde^1,2,4,5^, Philippe Pals^2,4^, Bob Asselbergh^1,2,3^, Roberto Dos Santos Dias^7^, Yannick Vermeiren^2,3^, Rik Vandenberghe^8^, Sebastiaan Engelborghs^2,3,9^, Peter Paul De Deyn^2,3,5^, Jean-Jacques Martin^2^, Patrick Cras^2,3,4^, Wim Annaert^7^, Christine Van Broeckhoven^1,2,3^, and BELNEU consortium.

1. Center for Molecular Neurology, VIB, Antwerp, Belgium.
2. Institute Born-Bunge, Antwerp, Belgium.
3. University of Antwerp, Antwerp, Belgium.
4. Department of Neurology, University Hospital Antwerp, Edegem, Belgium.
5. Department of Neurology, Hospital Network Antwerp, Antwerp, Belgium.
6. Department of Neurology, University Hospital Ghent and University of Ghent, Ghent, Belgium.
7. Center for Brain and Disease Research VIB, and Department of Neurosciences KU Leuven, Leuven, Belgium.
8. Department of Neurology, University Hospitals Leuven and Department of Neurosciences, KU Leuven, Leuven, Belgium.
9. Department of Neurology, UZ Brussel and Center for Neurosciences, Vrije Universiteit Brussel (VUB), Brussels, Belgium.

#### Abstract

Dementia with Lewy bodies (DLB) and Parkinsons disease (PD) are clinically, pathologically and etiologically disorders embedded in the Lewy body disease (LBD) continuum,1 characterized by neuronal -synuclein pathology. Rare homozygous and compound heterozygous premature termination codon (PTC) mutations in the Vacuolar Protein Sorting 13 homolog C gene (VPS13C) are associated with early-onset recessive PD. We observed in two siblings with early-onset age (<45) and autopsy confirmed DLB, compound heterozygous missense mutations in VPS13C, p.Trp395Cys and p.Ala444Pro, inherited from their healthy parents in a recessive manner. In lymphoblast cells of the index patient, the missense mutations reduced VPS13C expression by 90% (p = 0.0002). Subsequent, we performed targeted resequencing of VPS13C in 844 LBD patients and 664 control persons. Using the optimized sequence kernel association test, we obtained a significant association (p = 0.0233) of rare VPS13C genetic variants (minor allele frequency 1%) with LBD. Among the LBD patients, we identified one patient with homozygous missense mutations and three with compound heterozygous missense mutations in trans position, indicative for recessive inheritance. In four patients with compound heterozygous mutations, we were unable to determine trans position. The frequency of LBD patient carriers of proven recessive compound heterozygous missense mutations is 0.59% (5/844). In autopsy brain tissue of two unrelated LBD patients, the recessive compound heterozygous missense mutations reduced VPS13C expression. Overexpressing of wild type or mutant VPS13C in HeLa or SH-SY5Y cells, demonstrated that the mutations p.Trp395Cys or p.Ala444Pro, abolish the endosomal/lysosomal localization of VPS13C. Overall, our data indicate that rare missense mutations in VPS13C are associated with LBD and recessive compound heterozygous missense mutations might have variable effects on the expression and functioning of VPS13C. We conclude that comparable to the recessive inherited PTC mutations in VPS13C, combinations of rare recessive compound heterozygous missense mutations reduce VPS13C expression and contribute to increased risk of LBD.

## VPS13 proteins in cell processes

### Targeting VPS13 proteins to membrane contact sites

Samantha K. Dziurdzik^1^, Michael Davey^1^ and Elizabeth Conibear^1^

1. Department of Medical Genetics, University of British Columbia, Vancouver, Canada.

#### Abstract

Mutations in each of the four human VPS13 (VPS13A-D) proteins are associated with distinct neurological disorders: Chorea acanthocytosis, Cohen syndrome, early-onset Parkinsons disease and spastic ataxia. Recent evidence suggests that the different VPS13 paralogs transport lipids between organelles at different membrane contact sites. However, how each VPS13 isoform is targeted to these different sites is not known. Yeast has a single Vps13 protein whose localization depends on developmental stage or nutrient conditions. We have found that the membrane localization of yeast Vps13 requires a conserved six-repeat region, the Vps13 Adaptor Binding (VAB) domain, which binds to organelle-specific adaptors. Our results suggest that all adaptors compete for a single binding site in the VAB domain. Using a systematic mutagenesis strategy to define the contribution of each repeat, we have identified the putative adaptor binding site. Importantly, missense disease-causing mutations are predicted to impact this binding site, suggesting a conserved adaptor binding role for the VAB domain. Current efforts are focused on identifying novel VAB binding partners in both yeast and humans.

### VPS13, the founding member of a protein family that mediates bulk lipid transfer at intracellular membrane contact sites

Marianna Leonzino^1,2,3,*^, Karin Reinisch^2^ and Pietro De Camilli^1,2,3^

1. Department of Neuroscience, Yale University School of Medicine, New Haven, CT, USA.
2. Department of Cell Biology, Yale University School of Medicine, New Haven, CT, USA.
3. Howard Hughes Medical Institute, Yale University School of Medicine, New Haven, CT, USA.

* Present address: CNR, Institute of Neuroscience at Humanitas Research Hospital, Milan, Italy.

#### Abstract

The VPS13 protein family has been implicated in a variety of cellular function. While in yeast all these functions are performed by a single VPS13 gene, mammalian genomes encode 4 different VPS13 proteins whose physiological roles differ and whose mutations result in distinct, generally severe, clinical conditions. However, as our recent studies suggest, the shared core function of all these proteins, and of the related protein ATG2, is to act as conduits for the bulk flow of lipids at sites of contacts between intracellular membranes. They do so by acting as bridges that tether two adjacent membranes and allow lipid flux along a hydrophobic groove that spans their entire length. Two of the mammalian VPS13 isoforms, VPS13A and VPS13D, whose loss-of-function mutations cause Chorea Acanthocytosis and heterogeneous movement disorders including ataxias, respectively, act at contacts between the ER and mitochondria, although they bind to mitochondria via different mechanisms. Such localization is consistent with the redundant role of yeast VPS13 and ERMES, a protein complex that mediates lipid transport between the ER and mitochondria, even though yeast VPS13 itself populates contact sites other than those between ER and mitochondria (PMID: 26370498; PMID: 27280386). Conserved and redundant roles of VPS13 proteins in mitochondria function underscore the dependence of these organelles, which are excluded from membrane traffic, on protein mediated lipid exchange with other membranes. The two other mammalian VPS13 isoforms, VPS13B and VPS13C, have a different subcellular localization, with VPS13C, whose mutations result in Parkinsons disease,1 acting at contacts between the ER and late-endosomes/lysosomes. In my talk, I will summarize these studies and discuss how these discoveries shed light on mechanisms of disease, with emphasis on Chorea Acanthocytosis.

Kumar, Leonzino Reinisch & De Camilli, JCB, 2018

PeiQi & Reinisch, JCB, 2020

Guilln-Samander, Leonzino & De Camilli, BioRxiv 2020

### Vps13 is associated with multiple organelles and is required for timely removal of bulky cellular remnants

Ody C.M. Sibon^1^

1. Department of Biomedical Sciences of Cells and Structures, University Medical Center Groningen, University of Groningen, The Netherlands.

#### Abstract

The evolutionary conserved VPS13A gene is associated with the neurodegenerative disorder Chorea Acanthocytosis. We demonstrated that VPS13A is a peripheral membrane protein, associated with mitochondria, the endoplasmic reticulum, and lipid droplets. VPS13A is localized at sites where the endoplasmic reticulum and mitochondria are in close contact. VPS13A interacts with the ER residing protein VAP-A via its FFAT domain. Interaction with mitochondria is mediated via its C-terminal domain. In VPS13A-depleted cells, ER and mitochondria contact sites are decreased, mitochondria are fragmented and mitophagy is decreased. VPS13A also localizes to lipid droplets and affects lipid droplet motility. We further explored possible cellular functions of VPS13A using Drosophila melanogaster with its large cells and versatile genetic tools as a model organism. We demonstrated the presence of increased numbers of lipid droplets in specific brain areas of Vps13 mutants. We also demonstrated that Drosophila Vps13 is required for timely removal of large cellular remnants. During this process a Vps13-rich and Vps13-dependent membranous structure is being formed that surrounds the to-be-degraded cellular remnants. We will discuss how these new data link impairment of Vps13-related proteins with neurodegeneration.

### The role of VPS13A in endolysosomal and autophagic pathways: a CRISPR/Cas9-based cellular model of ChAc for phenotype-based compound screening

Alba Tornero-cija^1^, Mara-ngeles Navas^2^, Olivier Vincent^1^ and Ricardo Escalante^1^

1. Instituto de Investigaciones Biomdicas Alberto Sols; C.S.I.C./U.A.M.; Madrid, Spain.
2. Universidad Complutense de Madrid. Spain.

#### Abstract

VPS13A is a lipid transfer protein that localizes to different membrane contact sites between organelles and its mutation causes the rare disease chorea-acanthocytosis (ChAc). Previous work from our laboratory demonstrated that VPS13A localizes at the interface between mitochondria-endoplasmic reticulum and between mitochondria-endosomes in HeLa cells. Inhibition of VPS13A expression by siRNAs results in defects in lysosome function and accumulation of endolysosomal markers such as RAB7A and LAMP1 (Muoz-Braceras, S; Tornero-cija, AR; Vincent, O and Escalante, R. (2019). VPS13A is closely associated with mitochondria and is required for efficient lysosomal degradation. Dis Model Mech. 12(2)(PMID: 30709847). Accumulation of endolysosomal markers could be a useful phenotype for testing compounds in preclinical studies. However, the high cellular variability intrinsically linked to the use of siRNAs prevented us from obtaining conclusive results. We have optimized a CRISPR/Cas9 approach to generate a reliable model to compare wild-type and VPS13A Knock-out HeLa cells. The characterization of this model and preliminary results will be presented.

### Towards understanding the function of VPS13B in vesicular trafficking through the study of spermiogenesis

Romain Da Costa^1,2*^, Morgane Bordessoules^1,2^, Magali Guilleman^3^, Virginie Carmignac^1,4^, Hortense Courot^1^, Amandine Bataille^5^, Amandine Chlmaire^5^, Cline Bruno^1,3^, Patricia Fauque^1,3^, Christel Thauvin^1,2,6^, Laurence Faivre^1,2,7^ and Laurence Duplomb^1,2^

1. Inserm, UMR1231, Equipe GAD, Universit de Bourgogne Franche Comt, F-21000 Dijon, France.
2. FHU TRANSLAD, CHU Dijon, F-21000 Dijon, France.
3. Laboratoire de Biologie de la Reproduction, Hpital Franois Mitterrand, Universit de Bourgogne, F-21000 Dijon, France.
4. Centre de Rfrence Maladies Gntique Expression Cutane MAGEC-Mosaique, CHU Dijon, Dijon France.
5. Plateforme dImagerie Cellulaire CellImaP/DimaCell, Inserm LNC UMR1231, F-21000 Dijon, France.
6. Centre de Rfrence Dficiences Intellectuelles de Causes Rares, CHU Dijon, F-21000 Dijon, France.
7. Centre de Rfrence Anomalies du Dveloppement et Syndromes Malformatifs, CHU Dijon, F-21000 Dijon, France.

* *romain.dacosta@chu-dijon.fr*

#### Abstract

Cohen syndrome (CS) is a rare genetic disorder caused by variations affecting the VPS13B gene. It is characterized by a wide variety of clinical features that includes a typical facial dysmorphism, hypotonia, neutropenia, microcephaly, intellectual disability, and severe visual impairments. Understanding the molecular function of VPS13B is a prerequisite to the development of therapeutic approaches to treat chronic and progressive symptoms of CS. So far, very little is known about VPS13B cellular functions. Based on its domain structure and homologies with the yeast VPS13, VPS13B is likely to play a role in vesicle-mediated sorting and transport of proteins. To study these functions, we produced the Vps13bEx3/Ex3 mouse model. Initial phenotyping of the line revealed that male mutant mice were infertile due to morphologically abnormal, non-motile and short-lived spermatozoa. Histological analysis of the spermiogenesis showed that Vps13bEx3/Ex3 spermatids were unable to produce an acrosome, the large nuclear-capping vesicle that contains the hydrolases and other components allowing the sperm nucleus entry into the oocyte. In accordance with previous reports on patient fibroblasts we found that the Golgi apparatus was affected in Vps13bEx3/Ex3 spermatids. It lacked the ability to orientate towards the nucleus and surround the nuclear membrane. In addition, marking Golgi-derived vesicles with a lectin (PNA) revealed that instead of being targeted to the nuclear membrane those vesicles were targeted to the early endosome of early spermatids. As spermatids matured, acrosomal vesicles were found enclosed and degraded in lysosomal structures. Altogether, our study shows that Vps13b is an acrosomal membrane protein that redirects Golgi-derived vesicles towards the nuclear membrane. We now investigate whether VPS13B is also an addressing factor for other subsets of vesicles in CS-affected tissues and try to identify the compartments it targets.

### Functional insights from structural studies of VPS13s and related proteins

Karin M. Reinisch^1^

1. Department of Cell Biology, Yale School of Medicine, 333 Cedar Street, New Haven, CT, 06520, USA.

#### Abstract

Insights as to VPS13 function from structural and biochemical studies will be discussed. We found that VPS13 and related proteins may form bridges between membranes of organelles apposed at so-called contact sites that allow for bulk transfer of lipids between them. In this model, VPS13 proteins transfer lipids between the cytosolic leaflets of these membranes. In order to avoid bilayer asymmetry as lipids are depleted or augmented in just one of the leaflets of the donor and acceptor membrane, VPS13 proteins likely cooperate with scramblases that equilibrate lipids between bilayer leaflets.

## VPS13 proteins in health and disease

### Using human disease mutations to dissect the functions of yeast Vps13

Jae-Sook Park^1^ and Aaron M. Neiman^1^

1. Dept. of Biochemistry and Cell Biology, Stony Brook University, USA

*Aaron.Neiman@stonybrook.edu*

#### Abstract

The large size of the Vps13 protein makes screening for functionally significant missense mutations difficult. Missense alleles identified by sequencing of patients with VPS13-related diseases in humans, therefore, provide a valuable resource to explore structure-function relationships of this protein family. We have introduced mutations in the yeast VPS13 based on alleles found in chorea-acanthocytosis (VPS13A), Cohen syndrome (VPS13B), FTLD (VPS13C), and cerebellar ataxia (VPS13D) patients. Collectively, these mutations occur in different domains across the protein.

Alleles from chorea-acanthocytosis and cerebellar ataxia patients create distinct separation-of-function phenotypes that can be linked to changes in the steady-state distribution of the protein. The combination of either of these alleles with a second, intragenic mutation in VPS13 restores localization and suppresses the mutant phenotypes. The implications of this genetic interaction for the regulation of yeast Vps13 will be discussed.

### Vps13 protein and calcium signaling in yeast

Joanna Kaminska^1^, Piotr Soczewka^1^, Damian Kolakowski^1^, Patrycja Wardaszka^1^ and Teresa Zoladek^1^

1. Dept. of Biochemistry and Cell Biology, Stony Brook University, USA

#### Abstract

Vps13 are lipid transfer proteins localized in several membrane contact sites, such as nuclear-vacuolar junctions and endosome-mitochondrial junctions. Defects in Vps13(A-D) proteins in humans result in a rare neurodegenerative disease of unknown pathogenesis for which there is no cure. Yeast is a good model system to study function and regulation of Vps13 proteins, the effect of human mutations on cell physiology and to screen for genetic and chemical suppressors of vps13 mutations. In yeast, there is a unique VPS13 gene and its deletion, vps13, impairs intracellular trafficking and the actin cytoskeleton, and renders cells hypersensitive to various stress conditions, including sodium dodecyl sulphate (SDS) stress. Using this phenotype we found that vps13 is suppressed by expression of MYO3 fragment encoding N-terminus of myosin which binds calmodulin, RCN2 encoding a negative regulator of calcineurin, a calcium-dependent phosphatase, PMC1 gene for vacuolar calcium transporter, and mutant gene encoding calmodulin. We also found that calcium signaling is increased and calcineurin is hyperactivated in vps13 mutant. Importantly, ethylene glycol tetraacetic acid (EGTA), which sequesters calcium, and FK506 inhibitor of calcineurin were found to be suppressors of vps13 growth defect, pointing to the possible target for chemical intervention in VPS13-related diseases. Furthermore, we found that APT1 domains from yeast Vps13 and human Vps13A bind specific phospholipids and this binding is calcium-dependent. Pathogenic mutation in APT1 domain of Vps13A diminished phospholipid binding and changed its regulation. This supports the view that calcium regulation is also important for functioning of Vps13 proteins and in pathogenesis of VPS13-related diseases. This study was financed by the National Science Centre, Poland (UMO-2015/19/B/NZ3/01515).

### Therapeutic Targeting of Lyn Kinase To Treat ChoreaAcanthocytosis: evidence from pre-clinical studies

Kevin Peikert^1,19^, Enrica Federti^2^, Alessandro Matte^2^, Gabriela Constantin^2^, Enrica Caterina Pietronigro^2^, Paolo Francesco Fabene^3^, Paola Defilippi^4^, Emilia Turco^4^, Federico Del Gallo^3^, Piero Pucci^5,21^, Angela Amoresano^5^, Anna Illiano^5^, Flora Cozzolino^5,21^, Maria Monti^5,21^, Francesca Garello^6^, Enzo Terreno^6^, Seth L. Alper^7^, Hannes Gla^1^, Lisann Pelzl^8,9^, Katja Akgn^10^, Tjalf Ziemssen^10^, Rainer Ordemann^11^, Florian Lang^8^, Anna Maria Brunati^12^, Elena Tibaldi^12^, Immacolata Andolfo^13^, Achille Iolascon^13^, Mario Buffelli^3^, Carlo Zancanaro^3^, Erika Lorenzetto^3^, Angela Siciliano^2^, Massimiliano Bonifacio^2^, Adrian Danek^15^, Ruth H Walker^16,17^, Andreas Hermann^1,18,19,18,20*^ and Lucia De Franceschi^2^

1. Translational Neurodegeneration Section Albrecht-Kossel, Department of Neurology, University Medical Center Rostock, University of Rostock, Rostock, Germany.
2. Department of Medicine, University of Verona; Verona; Italy.
3. Dept of Neuroscience, Biomedicine and Movement Sciences, University of Verona; Verona; Italy.
4. Department of Biotecnologie Molecolari e Scienze per la Salute, University of Torino; Italy.
5. Dept of Chemical Sciences, University Federico II of Napoli, Napoli; Italy.
6. Molecular Imaging Center Dept. of Molecular Biotechnology and Health Sciences, University of Torino; Italy.
7. Division of Nephrology, Beth Israel Deaconess Medical Center, Harvard Medical School, Boston (MA), USA.
8. Department of Physiology I, Eberhard Karl University, Tbingen, Germany.
9. Transfusion Medicine, Eberhard Karl University, Tbingen, Germany.
10. Department of Neurology, University Hospital Carl Gustav Carus, Technische Universitt Dresden, Dresden, Germany.
11. Medical Department I, University Hospital Carl Gustav Carus, Technische Universitt Dresden, Dresden, Germany.
12. Dept of Molecular Medicine, University of Padua, Padua, Italy.
13. Dept. of Molecular Medicine and Medical Biotechnology; University of Naples Federico II, Naples, Italy.
14. Department of Neurology, James J. Peters Veterans Affairs Medical Center, Bronx (NY) USA.
15. Department of Neurology, Ludwig Maximilians University of Munich; Munich; Germany.
16. Department of Neurology, Icahn School of Medicine at Mount Sinai, New York, USA.
17. DZNE, German Center for Neurodegenerative Diseases, Research Site Dresden and Rostock/Greifswald, Germany.
18. Center for Transdisciplinary Neurosciences Rostock (CTNR), University Medical Center Rostock, University of Rostock, Rostock, Germany.
19. Division for Neurodegenerative Diseases, Department of Neurology, Technische Universitt Dresden, Dresden, Germany.
20. Center for Regenerative Therapies Dresden, Dresden; Germany.
21. CEINGE Biotecnologie Avanzate, Naples, Italy.

#### Abstract

Chorea-Acanthocytosis (ChAc) is a devastating, little understood, and currently untreatable neurodegenerative disease caused by VPS13A mutations. Based on our recent demonstration that accumulation of activated Lyn tyrosine kinase is a key pathophysiological event in human ChAc cells, we took advantage of Vps13a^-/-^ mice, which phenocopied human ChAc. Vps13a^-/-^ mice display (i) acanthocytes; (ii) signs of both hyper- and hypokinetic movement disorders; (iii) accumulation of active Lyn and of autophagy-related proteins in RBCs; and (iv) RBC retention of remnants of double1 membrane and multivesicular bodies. In isolated basal ganglia of Vps13a^-/-^ mice, we found signs of neurodegeneration associated with (i) accumulation of Lyn, stabilized in high molecular weight complexes; (ii) accumulation of autophagy related proteins; and (iii) reduction in expression of beclin-1, a key initiator of autophagy, due to increased caspase 3 activity. Noteworthy, we also found neuroinflammation in Vps13a^-/-^ mice associated with activation of NF-kB p65 and increased expression of IL-1b further emphasizing similarities between ChAc and other neurodegenerative disorders characterized by abnormal proteostasis such as PD or AD. Normalization of phenotypes in the Vps13a^-/-^ Lyn^-/-^ double knock out model substantiates the central role of accumulation of active Lyn in the pathophysiology of ChAc. Using proteomic approach, we found accumulation of, alpha-synuclein and phospho-tau proteins in Vps13a^-/-^ basal ganglia secondary to impaired autophagy leading to neuroinflammation. Mice double knockout Vps13a^-/-^ Lyn^-/-^ showed normalization of red cell morphology and improvement of autophagy in basal ganglia. We then in vivo tested pharmacologic inhibitors of Lyn: Dasatinib and Nilotinib. Dasatinib failed to cross the mouse brain blood barrier (BBB), but the more specific Lyn kinase inhibitor nilotinib, crosses the BBB. Nilotinib ameliorates both Vps13a^-/-^ hematological and neurological phenotypes, improving autophagy and preventing neuroinflammation. Our data support the repurposing of nilotinib as new therapeutic option for ChAc patients to be tested in phase II clinical trial.

### VPS13A distribution in the mouse basal ganglia

Esther Garca-Garca^1,2,3^, Albert Coll-Manzano^1,2,3^, Maria Carreras-Caball^1,2,3^, Nerea Chaparro-Cabanillas^1,2,3^, Merc Masana^1,2,3^, Jordi Alberch^1,2,3^ and Manuel J. Rodrguez^1,2,3^

1. Dept. Biomedical Sciences, Institute of Neurosciences, School of Medicine and Health Sciences, Universitat de Barcelona, Barcelona, Spain
2. Institut dInvestigacions Biomdiques August Pi i Sunyer (IDIBAPS), Barcelona, Spain
3. Centro de Investigacin Biomdica en Red sobre Enfermedades Neurodegenerativas (CIBERNED), Madrid, Spain.

#### Abstract

Chorea-acanthocytosis (ChAc) is caused by a VPS13A gene mutation leading to marked reduction or absence of VPS13A protein. ChAc patients show progressive movement disorders such as chorea, dystonia, parkinsonism and involuntary oral biting, among others. Since progressive impairment of basal ganglia circuitry underlie the appearance of movement disorders, it has been proposed that dysfunction of basal ganglia may account for ChAc pathophysiology. Actually, one of the main neuropathologic features in VPS13A mutations is a selective degeneration of the brain striatum. Because of that, a complete characterization of both VPS13A function and ChAc neuropathology in the brain and specifically in striatal neurons is needed. However, there is a poor knowledge about the VPS13A expression in the brain. We analyzed the VPS13A distribution in the mouse basal ganglia and related nuclei. We found a widespread VPS13A distribution along basal ganglia, with low expression levels in the striatum. By contrast we found higher VPS13A expression in the cortex. Thalamic nuclei and substantia nigra presented moderate VPS13A expression. VPS13A was present in glutamatergic pyramidal neurons, but also in some GABAergic neurons. At the subcellular level VPS13A was located in the soma and neurites, interacting with both the endoplasmic reticulum and mitochondria. However, we found no VPS13A within the dendritic spines by immunocytochemistry, which is consistent with the presence but not enrichment of VPS13A we found in cortical synaptosomes. These results indicate that VPS13A has not a direct role in synaptic transmission. However, the relatively low VPS13A expression in the striatum appears to be related with the specific vulnerability of this nucleus to ChAc. Thus, characterization of the molecular and cellular basis of this selective striatal cell loss should report significant progress toward the knowledge of ChAc pathophysiology.

Supported by SAF2017-88076-R, Ministerio de Ciencia, Innovacin y Universidades, and Fundacin ChAc.

### Chorein maintains mitochondrial morphology in mouse sperm and interacts with mitochondrial enzyme IDH3A

Kaoru Arai^1^, Omi Nagata^1^, Hitoshi Sakimoto^1^, Akira Sano^1^ and Masayuki Nakamura^1^

1. Department of Psychiatry, Kagoshima University Graduate School of Medical and Dental Sciences, 8-35-1 Sakuragaoka, Kagoshima, 8908520, Japan

#### Abstract

ChAc model mice, which have a homozygous deletion of exons 6061 in Vps13a, exhibited male infertility. They showed no significant differences from wild types in terms of sperm count, or sexual activity. They showed severely diminished sperm motility.

Immunocytochemical study revealed that chorein immunoreactivity is detected only in the mid-piece, mitochondria-rich region, of the sperm of wild type mice. Electron microscopy revealed abnormal ultrastructural morphology of the mitochondria in the mid-piece of sperm from ChAc model mice. The morphological abnormalities were seemed to occur at late stage of mitochondrial sheath developmental process in spermatogenesis. These results suggest that chorein is essential in mouse sperm for the arrangement of mitochondria and sperm motility. In addition, we identified isocitrate dehydrogenase 3 alpha sub unit (IDH3A), a mitochondrial tricarboxylic acid cycle enzyme as a chorein-interacting protein using proteomics analysis following co-immunoprecipitation (co-IP) of mouse sperm. Chorein-1DH3A interaction was also detected in both mouse striatum and Human Embryonic Kidney cells. Western blotting analysis revealed the reduction of immunoreactivities of the IDH3A in ChAc model mouse sperm. Although the relationship between IDH3A reduction and morphological abnormalities of mitochondria in ChAc model mouse remains unclear, it was suggested that chorein, mitochondrial morphology and IDH3A are involved in ChAc pathophysiology.

## Present and future clinical care of neuroacanthocytosis

### Old and novel clinical and paraclinical markers of Chorea acanthocytosis

Andreas Hermann^1,2,3^

1. Translational Neurodegeneration Section Albrecht-Kossel, Department of Neurology, University Medical Center Rostock, University of Rostock, Rostock, Germany.
2. DZNE, German Center for Neurodegenerative Diseases, Research Site Rostock/Greifswald, Rostock, Germany.
3. Center for Transdisciplinary Neurosciences Rostock (CTNR), University Medical Center Rostock, University of Rostock, Rostock, Germany.

#### Abstract

Chorea-Acanthocytosis (ChAc) is an ultra-rare neurodegenerative disease caused by mutations in the VPS13A gene. It is characterized by a spectrum of neurological symptoms (e.g., chorea, epilepsy, parkinsonism, cognitive decline or neuro-/myopathy) and acanthocytosis. It is thus not only a typical differential of Huntingtons disease, but also for other disease conditions. Thus, markers which help in the diagnostics of ChAc would be helpful. Furthermore, both pathognomonic markers and markers for clinical progression are of urgent need in case one want to perform clinical trials.

Even though possibly representing with a plethora of clinical phenotypes, there are some which clearly direct us to ChAc. E.g. clinical symptoms such as orolingual biting, tongue protrusions and loss of deep tendon reflexes are typically found in ChAc. Blood cell acanthocytosis clearly helps diagnosing ChAc, but also unexplained and persistent CK elevation independent from activity or seizures in the context of a movement disorder should make us think of ChAc. Of note, however, there is surprisingly little known about the longitudinal behavior during the disease course of all of these clinical and paraclinical biomarkers and thus their strength as potential read-outs parameters for disease modifying actions remains elusive.

The talk will give insights in classical and novel potential markers of ChAc. Limited knowledge on the natural history of ChAc remains a barrier for clinical trial readiness.

### Outcome of deep brain stimulation in two patients with chorea-acanthocytosis

Berta Pascual-Sedano^1,2,3^, J Prez-Prez^1^, L Rebordo^4^, C Garca-Snchez^1,3^, I Aracil^1,2,3^, A Campolongo^1,2,3^, A Gironell^1,3^, R Rodriguez-Rodrguez^5^, MJ lvarez- Holzapfel^5^, B Gmez- Ansn^6^, V Medina^6^, J Pagonabarraga^1,2^ and J Kulisevsky^1,2,3^

1. Movement Disorders Unit, Neurology department, Hospital de la Santa Creu I Sant Pau, Barcelona, Spain.
2. Faculty of Health Sciences, Universitat Oberta de Catalunya (UOC), Barcelona, Spain.
3. Center for Networked Biomedical Research in Neurodegenerative Diseases (CIBERNED), Instituto de Salud Carlos III, Madrid, Spain.
4. Department of Neurology, Hospital Professor Doutor Fernando Fonseca, Amadora, Portugal.
5. Neurosurgery Departament, Hospital de la Santa Creu i Sant Pau, Barcelona, Spain.
6. Neuroradiology, Hospital de la Santa Creu i Sant Pau, Barcelona, Spain.

#### Abstract

Chorea-acanthocytosis is an autosomal recessive very disabling disease characterized by multiorgan involvement, including central and peripheral nervous system. The available treatment is symptomatic. We present the outcome of two patients who underwent deep brain stimulation (DBS).

##### Case Reports

**Case 1:** A 30-year-old man with familiar history of bipolar disease and consanguinity, presented with seizures at 25 years old. One year later, progressive orofacial movements and vocalizations appeared. After that, he started with lingual mutilation, weight loss and behavior problems. He showed inattention, impulsivity, vocalizations, dysarthria, sialorrhea, feeding dystonia, generalized chorea, bradykinesia and instability with freezing. Genetic test was positive for chorea-acanthocytosis. He became partially dependent, with severe sleeping and eating impairment. UHDRS-TMS 51/124, UHDRS-FCS 8/13, UHDRS-IS 50%. Pallidal DBS was performed. Three months1 post-DBs he had a marked improvement of attention, gait, swallowing, weight and chorea, becoming almost independent (UHDRS-TMS 14/124, UHDRS-FCS 20/33, UHDRS-IS 70%). At 14 months post-surgery the improvement was maintained.

**Case 2:** 43-year-old man with chorea-acanthocytosis started at 21 years-old (biting the lower lip). He later suffered of progressive motor and behavioral involvement. Blood test confirmed acanthocytes, and corein was positive in red blood cells. Due to severe feeding dystonia, PEG was needed for enteral feeding. Over time, choreo-ballism prevented him from walking and even sitting due to falls, and he had serious lip mutilation, vocalizations, anxiety, disinhibition, lack of judgment and obsessions. Scores pre-DBS: UHDRS-TMS 52/124, UHDRS-FCS 5/13. Pallidal DBS was performed and just after DBS-activation he had a marked improvement, especially on feeding dystonia (he no longer uses PEG), choreo-ballism (reducing falls and allowing sitting) and behavior; this improvement is maintained 5 months post-DBS. However, a freezing of gait has appeared.

**Conclusion:** Functional surgery can be useful to improve motor and behavioral symptoms in patients with chorea-acanthocytosis; however, the eligibility should be individualized considering the benefit-risk profile.

### Targeting Lyn kinase and PI3K pathway Potential therapies for chorea-acanthocytosis in the future?

Kevin Peikert^1,2^, Hannes Gla^1,2^, Alessandro Matte^3^, Enrica Federti^3^, Lisann Pelzl^4,5^, Katja Akgn^2^, Tjalf Ziemssen^2^, Rainer Ordemann^6^, Florian Lang^4^, Lucia De Franceschi^3^ and Andreas Hermann^1,2,79^

1. Translational Neurodegeneration Section Albrecht-Kossel, Department of Neurology, University Medical Center Rostock, University of Rostock, Rostock, Germany.
2. Department of Neurology, University Hospital Carl Gustav Carus, Technische Universitt Dresden, Dresden, Germany.
3. Department of Medicine, University of Verona, Verona, Italy.
4. Department of Physiology I, University of Tbingen, Tbingen, Germany.
5. Transfusion Medicine, Medical Faculty, Eberhard Karl University, Tbingen, Germany.
6. Medical Department I, University Hospital Carl Gustav Carus, Technische Universitt Dresden, Dresden, Germany.
7. Center for Regenerative Therapies Dresden (CRTD), Technische Universitt Dresden, Dresden, Germany.
8. DZNE, German Center for Neurodegenerative Diseases, Research Site Rostock/Greifswald, Rostock, Germany.
9. Center for Transdisciplinary Neurosciences Rostock (CTNR), University Medical Center Rostock, University of Rostock, Rostock, Germany.

#### Abstract

Chorea-Acanthocytosis (ChAc) is an ultra-rare neurodegenerative disease caused by mutations in the VPS13A gene. It is characterized by a spectrum of neurological symptoms (e.g., chorea, epilepsy, cognitive decline or myopathy) and acanthocytosis. Elevated Lyn kinase activity and altered PI3K signaling has been identified as key pathophysiological mechanisms. They represent promising druggable targets for a potential disease-modifying therapy.

We evaluated an individual off-label treatment with the FDA-approved Lyn kinase inhibitor Dasatinib in three ChAc patients. Alongside with a thorough safety monitoring, we assessed clinical (e.g. UPDRS, UHDRS, Quality of Life) as well as paraclinical and experimental parameters (e.g. acanthocyte and creatine kinase level, Lyn kinase activity, actin cytoskeleton in red blood cells).

Dasatinib treatment was safe in all three patients. Only mild side effects occurred. The clinical parameters remained stable without significant improvement or deterioration. Regain of deep tendon reflexes was observed in one patient. Reduction of elevated Lyn kinase activity and autophagy markers as well as restoration of actin cytoskeleton was found in red blood cells.

In summary, the experimental read-outs point to target engagement in the red blood cells. However, effects on the central nervous system could not be shown within a rather short duration of treatment with the predefined read-outs.

We furthermore discuss the rationale and perspectives of Lithium treatment modifying downstream effects of altered PI3K signaling in ChAc.

### Neuroacanthocytosis case registry: current status and future goals

Adrian Danek^1^, Benedikt Bader^1,2^, Gabriel Miltenberger-Miltenyi^1,3^, Kevin Peikert^4^, Bernhard Landwehrmeyer^5^, Glenn and Ginger Irvine^6^

1. Neurologische Klinik und Poliklinik, Ludwig-Maximilians-Universitt Mnchen, Germany.
2. Schn Klinik Bad Aibling, Fachklinik fr Neurologie, Bad Aibling, Germany.
3. Instituto de Medicina Molecular, Faculdade de Medicina da Universidade de Lisboa, Portugal.
4. Klinik und Poliklinik fr Neurologie, Universittsmedizin Rostock, Germany.
5. Klinik fr Neurologie, Universittsklinikum Ulm, Germany.
6. Advocacy for Neuroacanthocytosis Patients, London, UK.

#### Abstract

Establishing a case registry was discussed from the very first symposium in 2002 to define the natural history of neuroacanthocytosis syndromes and to serve as recruitment tool for future therapeutic trials. Set up as a submodule of the European Huntingtons Disease Network (*www.euro-hd.net/html/na/registry*), it offers room for an extensive set of patient information. First data were deposited in 2008 and 50+ cases overall were entered. The registry was most active while part of the first EMINA consortium yet has been dormant since. It is a pressing need to revive the registry effort and to tackle open questions. Originally conceived for all neurocanthocytosis syndromes, it appears more appropriate to include only cases of chorea-acanthocytosis and of McLeod syndrome, both only with biologically confirmed diagnoses. Ethical and data protection issues must be re-analysed and amended accordingly. Consent forms so far were available only in English. Data monitoring and quality control must be implemented. In addition to clinical data there must be an opportunity to collect imaging data such as from MRI, but also data from wearables and other aspects of e-health are of interest. Data-entering colleagues had willingly collaborated but the motivation of busy clinicians must be improved. One possible incentive may be the free diagnostic information (chorein Western blot service) made available by the patient advocacy but also direct financial reimbursement could be considered. Case acquisition might be further improved trough a mechanism for self-registration by patients. Continuous contact and communication will be essential to obtain follow-up data.

The questions of data ownership, data sharing and of publication policies deserve serious effort.

Finally, to secure continuity after 2023 we plan a gradual transition of responsibility from the Munich to the Rostock group.

## Glenn Irvine prize awardee

### XK is a partner for VPS13A: A molecular link between Chorea Acanthocytosis and McLeod syndrome

Jae-Sook Park^1^ and Aaron M. Neiman^1^

1. Dept. of Biochemistry and Cell Biology, Stony Brook University, USA

#### Abstract

Vps13 is a highly conserved lipid transfer protein found at multiple inter-organelle membrane contact sites. In yeast, the single Vps13 protein is recruited to different contact sites through interaction with different adaptor proteins. Mutations in human VPS13A cause the neurodegenerative disease Chorea Acanthocytosis (ChAc). The symptoms of ChAc resemble those of McLeod syndrome caused by mutations in the XK gene. These observations suggest that XK could be a partner protein for VPS13A. We report that XK forms a complex with VPS13A in human cells, and XK overexpression relocalizes VPS13A from lipid droplets to subdomains of endoplasmic reticulum (ER). A VPS13A protein carrying the ChAc-linked mutation (W2460R) in the VPS13 adaptor binding (VAB) domain failed to localize to lipid droplets and did not relocalize to ER subdomains upon XK overexpression. These observations suggest that the function of VAB domain in regulating VPS13 localization might be conserved in human VPS13A and that disruption of a VPS13A-XK complex is the common basis for ChAc and McLeod syndrome.

## Focus is on patients, with caregivers present

### Impact of Neuroacanthocytosis syndromes on speech and swallowing function

E. Tripoliti^1,2^

1. Institute of Neurology, Unit of Functional Neurosurgery, UCL, London UK
2. National Hospital for Neurology and Neurosurgery, UCLH, NHS Trust, London UK.

#### Abstract

**Aim:** The aim of the talk was to describe the variability of speech and swallowing presentation in patients with NA syndromes and to discuss treatment options for behavioural therapies.

**Background:** Speech and communication can be affected at every stage of the disease process, and dysarthria can be one of the earlier symptoms of the disease. Similarly maintaining the safety and pleasure from eating and drinking with family is part of our quality of life.

**Methods:** A combination of anecdotal evidence from short reports and clinical experience shows the wide spectrum and the changes of presentation, from hyperkinetic (too much movement) to hypokinetic (too little). Symptoms affecting speech involve choreic movements of the trunk and the limbs, oro-facio-lingual dyskinesias, limb or facial dystonias, and difficulty initiating movement, with reduced amplitude. Factors contributing to the variability of presentation are age of onset (young adult or middle aged), other treatments (surgical, BOTOX, pharmacological), effect of stress and fatigue, and availability of multidisciplinary team input.

**Results:** The aim of any speech and swallowing intervention is to maintain communication and participation for as long as possible. A variety of speech approaches can be deployed, depending on the stage and the particular symptoms. Sensory tricks can help and need to be acknowledged and expanded. Mouth guards can be useful for tongue dystonia but can impair speech. BOTOX and other pharmacological effects should be monitored for their effects on function before and after their initiation. Working on louder voice can improve not just voice, but articulation clarity. Singing is always beneficial for both the voice and the soul. Voice banking and Alternative communication systems will be discussed.

Swallowing can be affected in the oral stage by the uncontrolled movements of the tongue and the reduced lip seal. Pharyngeal stage can preserve the safety of swallowing. Patients should be encouraged to sit more upright if possible and to minimize distractions during meals. Training the expiratory muscles (with e.g. the EMST150) can help with the cough strength and clearing of saliva.

**Conclusion:** Maintaining communication and swallowing function for as long as possible is paramount. Balancing the hypo-and hyper-kinetic symptoms throughout the disease process requires flexibility and further knowledge, through more longitudinal studies.

## Poster # 1

### Detection of PANK2 mutations in the B siblings whose Y2721C VPS13A lacks effects in cell models

Adrian Danek^1^, Jae-Sook Park^2^, Aaron Neiman^2^, Antonio Velayos-Baeza^3^, Gabriel Miltenberger-Miltenyi^1,4^, Matias Wagner^5^, Bettina Schmid^6^, Alexander Hruscha^6^, Qing Wang^1,6,7^, Richard Hardie^8^, Sonia Gandhi^9^ and Ginger Irvine^10^

1. Neurologische Klinik und Poliklinik, Ludwig-Maximilians-Universitt Mnchen, Germany.
2. State University of New York at Stony Brook, NY, USA.
3. Dept. of Physiology, Anatomy and Genetics/Wellcome Centre for Human Genetics University of Oxford, UK.
4. Instituto de Medicina Molecular, Faculdade de Medicina da Universidade de Lisboa, Portugal.
5. Technische Universitt Mnchen, Germany.
6. Deutsches Zentrum fr Neurodegenerative Erkrankungen, Munich, Germany.
7. Graduate School for Systemic Neuroscience, Ludwig-Maximilians-Universitt Mnchen, Germany.
8. Institute of Clinical Neurosciences, University of Bristol, UK.
9. National Hospital for Neurology and Neurosurgery, London, UK.
10. Advocacy for Neuroacanthocytosis Patients, London, UK.

#### Abstract

A sister and brother, aged 56 and 55, had been diagnosed with neuroacanthocytosis in their twenties, based on childhoodonset behavioural as well as movement abnormalities and on 15% acanthocytes (Hardie et al. 1991). Findings in the parents and family history were unremarkable. The original genetic work-up of chorea-acanthocytosis (ChAc) showed chromosome 9 linkage also in this CHAC9 pedigree (Rubio et al. 1997). When the responsible gene (CHAC, later renamed as VPS13A) was identified (Rampoldi et al. 2001), a single heterozygous missense mutation (c.8162A>G, Y2721C) was found in this family. The siblings clinical findings were, however, exceptional because of their onset age, normal reflexes and normal CK levels. Their mutation, along with missense mutations VPS13A L67P, I90K, A1095P, and I2771R was modelled in yeast (Park et al. 2016; Rzepnikowska et al. 2017). Among the cognate mutations (Vps13 L66P, C89K, L1107P, Y2702C, and I2749R) only Y2702C, corresponding to the CHAC9 mutation, failed to show a phenotype in yeast. In human-derived cells (HEK293T) we found the Y2721C mutant protein localizes like wild-type VPS13A protein. The siblings were thought lost to follow-up but after seeking contact with the Advocacy for Neuroacanthocytosis Patients they agreed to further analyses. Erythrocyte membrane Western blot showed a normally expressed VPS13A protein (chorein) band. Exome sequencing, confirming the c.8162A>G mutation, failed to find any other VPS13A mutations, yet detected heterozygous mutations in PANK2 (NM_024960.4): c.101T>A, F34Y and c.688G>A, G230R. We conclude that Y2721C is a benign VPS13A polymorphism and that the neuroacanthocytosis umbrella diagnosis in the siblings must be refined into pantothenate kinase associated neurodegeneration. These observations show that a diagnosis of ChAc must ideally be confirmed by reduced chorein expression and/or by proof of clearly pathogenic mutations in both VPS13A alleles. Mere presence of a single mutation with unclear functional effects is clearly insufficient to support the diagnosis

## Poster # 2

### Characterization of the distribution and localization of VPS13A in the mouse brain to understand the pathophysiology of chorea-acanthocytosis

Esther Garca-Garca^1,2,3^, Albert Coll-Manzano^1,2,3^, Maria Carreras-Caball^1,2,3^, Nerea Chaparro-Cabanillas^1,2,3^, Merc Masana^1,2,3^, Jordi Alberch^1,2,3^ and Manuel J. Rodrguez^1,2,3^

1. Dept. Biomedical Sciences, Institute of Neurosciences, School of Medicine and Health Sciences, Universitat de Barcelona, Barcelona, Spain
2. Institut dInvestigacions Biomdiques August Pi i Sunyer (IDIBAPS), Barcelona, Spain
3. Centro de Investigacin Biomdica en Red sobre Enfermedades Neurodegenerativas (CIBERNED), Madrid, Spain.

#### Abstract

Chorea-acanthocytosis (ChAc) is caused by VPS13A gene mutations leading to absence of the chorein protein. ChAc patients show adult-onset progressive movement disorders and a selective degeneration of the striatum. There is a poor knowledge about the localization and function of chorein in the brain. The objective of this work is to analyze the chorein distribution along the mouse brain and to evaluate the subcellular localization of chorein in cortical primary cultures. Thus, we evaluated the distribution of chorein mRNA and protein in mouse brain tissue by fluorescence in-situ hybridization and immunohistochemistry. This analysis showed a widespread distribution of chorein along the mouse brain, with different staining intensity profiles between nuclei. In general, the mRNA localization resembled that of the protein one with an enrichment in the pons and cerebellum. The hippocampus presented high chorein labelling in the pyramidal layer and in the granular layer of dentate gyrus. We found moderate staining in the cortex and in the most thalamic and hypothalamic nuclei. Interestengly, we found weak staining in the basal ganglia nuclei whereas we found no staining in white matter structures. Not only neurons but also some glial cells expressed chorein. Then, we evaluated the subcellular localization of chorein in cortical primary cultures by immunocytochemistry and in cerebral cortex by synaptosomal isolation. We found chorein in the soma but also in neurites, interacting with both the endoplasmic reticulum and mitochondria. However, we found no chorein staining within the dendritic spines of cortical neurons in culture. Finally, Chorein was present but no significantly enriched in the crude synaptosomal fraction of cerebral cortex tissue. Deciphering the chorein localization in the brain constitutes the first step to understand its role in the ChAc pathophysiology.

Funded by SAF2017-88076-R, Ministerio de Ciencia, Innovacin y Universidades and Fundacin ChAc.

## Poster # 3

### Normal chorein signal on red cell membrane Western blotting in VPS13A disease

Gabriel Miltenberger-Miltenyi^1^, Benedikt Bader^1^, Antonio Velayos-Baeza^2^, Lothar Burghaus^3^, Paul Goldsmith^4^, Angela Abicht^5^, Roongroj Bhidayasiri^6^, Nikhil Balakrishnan^7^, David Simon^8^, Ruth Walker^9^, Naomi Lubarr^9^ and Adrian Danek^1^.

1. Department of Neurology, Ludwig-Maximilians-Universitt Mnchen, Munich, Germany.
2. Wellcome Centre for Human Genetics, University of Oxford, Oxford, United Kingdom.
3. Klinik fr Neurologie, Heilig Geist-Krankenhaus, Kln, Germany.
4. Department of Neurology, Royal Victoria Infirmary, Newcastle Upon Tyne, UK.
5. Medical Genetics Center, Munich, Germany.
6. Chulalongkorn University, Bangkok, Thailand.
7. Neurosciences Institute, Neurology University, Charlotte, NC, USA
8. Department of Neurology, Beth Israel Deaconess Medical Center, Harvard Medical School, Boston, Massachusetts, USA.
9. Department of Neurology, Mount Sinai School of Medicine, New York, NY, USA.

#### Abstract

VPS13A disease is a rare, autosomal recessively inherited neurodegenerative disorder caused by mutations in the VPS13A gene, coding for the chorein protein. Next to DNA studies, Western blot (WB) analysis of chorein is of great diagnostic value for all suspected cases, in particular when quick diagnosis is required or when genetic studies of the complex VPS13A gene are not easily available. Dobson-Stone et al. (2004) showed that the chorein band detected by WB of erythrocyte (red blood cell, RBC) membranes was absent or severely reduced in VPS13A disease patients, with already confirmed VPS13A mutations. Financially supported by the Advocacy for Neuroacanthocytosis Patients (*www.naadvocacy.org*) we were able to offer the chorein WB for free as of 2006. Up to now we have received more than 700 samples from patients with a clinical suspicion of chorea-acanthocytosis. In 194 cases, we confirmed a diagnosis of VPS13A disease because of absence or clearly reduced levels of RBC membrane chorein, using a polyclonal antiserum (anti-chor1) against the N-terminal region of chorein (Dobsone-Stone et al. 2004). Interestingly, a number of patients showing normal chorein levels presented with highly suggestive clinical features of VPS13A disease; a genetic follow-up has been started in a number of cases and, so far, pathogenic mutations in VPS13A have been detected in six of them.

Our observations confirm that the chorein WB has less than 100% sensitivity for the diagnosis of VPS13A disease. Thus, they stress the need to follow up on patients with clinical features highly suggestive of VPS13A disease even if chorein WB appeared normal. These observations may relate to properties of the antibody used. Quantitative analyses of the chorein bands are pending. Studying these cases can provide valuable insight about functional domains and stability of chorein

## Poster # 4

### Novel mutations and findings in a large cohort of McLeod neuroacanthocytosis patients

Kevin Peikert^1^, Beate Schlotter-Weigel^2^; Federica Montagnese^2^, Peter Reilich^2^, Carsten Saft^3^, Franz Marxreiter^4,5^, Zacharias Kohl^6^, Stefan Evers^7,8^, Wolfgang von Kalckreuth^9^, Carsten Buhmann^10^, Beate Mayer^11^, Ernst Walther^12^, Armin Orth^13^, Manfred Hoenig^14^, Krassen Nedeltchev^15^, Wolfgang N Lscher^16^, Hans H Jung^17^, Maja Mattle-Greminger^18^, Beat M. Frey^18^, Andreas Hermann^1,19,20^ and Adrian Danek^21^.

1. Translational Neurodegeneration Section Albrecht-Kossel, Department of Neurology, University Medical Center Rostock, University of Rostock, Rostock, Germany.
2. Friedrich-Baur-Institut, Neurologische Klinik, Ludwig-Maximilians-Universitt Mnchen, Germany.
3. Department of Neurology, St. Josef Hospital, Gudrunstrae 56, 44791 Bochum, Germany.
4. Department of Molecular Neurology, University Hospital Erlangen, Schwabachanlage 6, 91054 Erlangen
5. Center for Rare Movement Disorders, University Hospital Erlangen, Schwabachanlage 6, 91054 Erlangen
6. Department of Neurology, University of Regensburg, Regensburg, Germany.
7. Medical Faculty, University of Mnster, Mnster, Germany.
8. Department of Neurology, Lindenbrunn Hospital, Coppenbrgge, Germany.
9. Praxis fr Neurologie und Psychiatrie an der Johanneskirche, Freiburg, Germany.
10. Department of Neurology, University Medical Center Hamburg-Eppendorf, Martinistrae 52, 20246 Hamburg, Germany.
11. Institute of Transfusion Medicine, Charit Universittsmedizin Berlin, corporate member of Freie Universitt Berlin, Humboldt-Universitt zu Berlin, and Berlin Institute of Health, Berlin, Germany.
12. Zentrum fr Neurologie und Neurorehabilitation, Schn Klinik Hamburg Eilbek, Hamburg, Germany.
13. Klinik fr Neurologie, Knappschaftsklinikum Saar, Pttlingen, Germany.
14. Department of Pediatrics, University Medical Center Ulm, Ulm, Germany.
15. Department of Neurology, Kantonsspital Aarau, Aarau, Switzerland.
16. Department of Neurology, Medical University of Innsbruck, Anichstrasse 35, 6020, Innsbruck, Austria.
17. Departement of Neurology, University Hospital and University of Zurich, Zurich, Switzerland.
18. Blood Transfusion Service Zrich, Swiss Red Cross (SRC), Zurich-Schlieren, Switzerland.
19. DZNE, German Center for Neurodegenerative Diseases, Research Site Rostock/Greifswald, Rostock, Germany.
20. Center for Transdisciplinary Neurosciences Rostock (CTNR), University Medical Center Rostock, University of Rostock, Rostock, Germany.
21. Neurologische Klinik und Poliklinik, Ludwig-Maximilians-Universitt Mnchen, Germany.

#### Abstract

McLeod syndrome (MLS) is an ultra-rare neurodegenerative X-linked disease caused by mutations in the XK gene, classified as one of the core neuroacanthocytosis syndromes. This is a retrospective and prospective analysis of genotype and phenotype of sixteen cases. We longitudinally characterized the second largest cohort published up to date and identified novel mutations in the XK gene and a novel contiguous gene deletion including the PRRG1 gene. Furthermore, we describe two cases with contiguous gene deletions including the XK and CYBB gene leading to X-linked chronic granulomatous disease in the childhood. This study confirms core features of MLS such as combination of movement disorder with neuro/myopathy and neuropsychiatric impairment, overall late onset, cardiac involvement, hyper-CK-emia, but describes obstructive sleep apnea and childhood occurrence of epileptic seizures as potentially novel aspects. Our study expands the limited knowledge on the variable course of the disease, different clinical phenotypes and the genetic spectrum.

## Poster # 5

### Targeting copper homeostasis improves functioning of vps13. yeast mutant cells, a model of VPS13-related diseases

Piotr Soczewka^1^, Dborah Tribouillard-Tanvier^2,3^, Jean-Paul di Rago^2^, Teresa Zoladek^1^, Joanna Kaminska^1^

1. lnstitute of Biochemistry and Biophysics Polish Academy of Science, 02-106 Warsaw, Poland.
2. IBGC, UMR 5095, CNRS, Universil de Bordeaux, F-33000 Bordeaux, France.
3. Institut National de la Sant et de la Recherche Mdicale (INSERM),F-33077 Bordeaux, France.

#### Abstract

Ion homeostasis is crucial for organism functioning, and its alterations may cause diseases. For example, copper insufficiency and overload are associated with Menkes and Wilsons diseases, respectively, and iron imbalance is observed in Parkinsons and Alzheimers diseases. To better understand human diseases, *Saccharomyces cerevisiae* yeast are used as a model organism. In our studies, we used the vps13D yeast strain as a model of rare neurological diseases caused by mutations in VPS13A-D genes. In this work, we show that overexpression of genes encoding copper transporters CTR1, CTR3, and CCC2, or the addition of copper salt to the medium, improved functioning of the vps13D mutant. We show that their mechanism of action, at least partially, depends on increasing iron content in the cells by the copper-dependent iron uptake system. Finally, we present that treatment with copper ionophores, disulfiram. elesclomol, and sodium pyrithione, also resulted in alleviation of the defects observed in vps13D cells. Our study points at copper and iron homeostasis as a potential therapeutic target for further investigation in higher eukaryotic models of VPS13-related diseases.

Soczewka, P.; Tribouillard-Tanvier, D.; di Rago, J.-P.; Zoladek, T.; Kaminska, J. lnt. J. Mol. Sci. 2021, 22, 2248. *https://doi.org/10.3390/ijms22052248*

